# Over 16% efficiency organic photovoltaic cells enabled by a chlorinated acceptor with increased open-circuit voltages

**DOI:** 10.1038/s41467-019-10351-5

**Published:** 2019-06-07

**Authors:** Yong Cui, Huifeng Yao, Jianqi Zhang, Tao Zhang, Yuming Wang, Ling Hong, Kaihu Xian, Bowei Xu, Shaoqing Zhang, Jing Peng, Zhixiang Wei, Feng Gao, Jianhui Hou

**Affiliations:** 1 State Key Laboratory of Polymer Physics and Chemistry, Beijing National Laboratory for Molecular Sciences, CAS Research/Education Center for Excellence in Molecular Sciences (BNLMS), Institute of Chemistry, Chinese Academy of Sciences, 100190 Beijing, China; 2 University of Chinese Academy of Sciences, 100049 Beijing, China; 3 Key Laboratory of Nanosystem and Hierarchical Fabrication, National Center for Nanoscience and Technology, 100190 Beijing, China; 4 Department of Physics Chemistry and Biology, Linköping University, SE-581 83 Linköping, Sweden; 5 School of Chemistry and Biological Engineering, University of Science and Technology Beijing, 100083, Beijing, China; 6 Organtec Ltd., 102200 Beijing, China

**Keywords:** Chemistry, Materials chemistry, Chemical synthesis, Optics and photonics

## Abstract

Broadening the optical absorption of organic photovoltaic (OPV) materials by enhancing the intramolecular push-pull effect is a general and effective method to improve the power conversion efficiencies of OPV cells. However, in terms of the electron acceptors, the most common molecular design strategy of halogenation usually results in down-shifted molecular energy levels, thereby leading to decreased open-circuit voltages in the devices. Herein, we report a chlorinated non-fullerene acceptor, which exhibits an extended optical absorption and meanwhile displays a higher voltage than its fluorinated counterpart in the devices. This unexpected phenomenon can be ascribed to the reduced non-radiative energy loss (0.206 eV). Due to the simultaneously improved short-circuit current density and open-circuit voltage, a high efficiency of 16.5% is achieved. This study demonstrates that finely tuning the OPV materials to reduce the bandgap-voltage offset has great potential for boosting the efficiency.

## Abstract (web summary)

Halogenation has proved an effective strategy to improve the power conversion efficiencies of organic solar cells but it usually leads to lower open-circuit voltages. Here, Cui et al. unexpectedly obtain higher open-circuit voltages and achieve a record high PCE of 16.5% by chlorination.

## Introduction

As a promising solar energy-harvesting technology, organic photovoltaic (OPV) cells have advantages like light-weight, flexibility, transparency, and potential low costs^1–3^. In the last three decades, great efforts have been devoted to material design, device engineering, morphology optimization, and mechanism study, contributing to the increase in the power conversion efficiencies (PCEs) from solar energy to electricity^4–20^. At present, although PCEs exceeding 15% have been achieved in single-junction OPV cells^21,22^, further improvement is still needed to compete with other photovoltaic technologies, such as silicon solar cells and perovskite solar cells.

Designing low bandgap materials to have a good match with the solar spectrum is a general method for improving the short-circuit current density (*J*_SC_) and thereby the PCEs of OPV cells^23–28^. In the last few years, the development of low bandgap non-fullerene small molecular acceptors with acceptor–donor–acceptor structures has achieved great success^29–33^. Halogenation of electron-accepting units can enhance the intramolecular charge transfer (ICT) effects and reduce the bandgaps of non-fullerene small molecular acceptors, which has been demonstrated to be one of the most effective molecular design strategies^34–36^. To date, most of the top-performing acceptors, such as IT-4F^36^, IEICO-4F^37^, and BT-CIC^13^, contain fluorine or chlorine atoms.

As chlorination is easier in the synthesis and plays a more pronounced role in enhancing the ICT effect than fluorination does, it is more attractive for designing highly efficient OPV materials^38–41^. However, one of the biggest problems of chlorinated acceptors is that they usually exhibit downshifted lowest unoccupied molecular orbit (LUMO) levels, leading to reduced open-circuit voltages (*V*_OC_s) in the resulting OPV cells. For example, a 100 meV downshift of the LUMO level and 40 nm red-shift of the absorption spectrum were observed when the fluorine atoms in IT-4F were replaced by the chlorine atoms of IT-4Cl^40^. The IT-4Cl-based OPV cell yielded an increased *J*_SC_ with significant sacrifice of *V*_OC_. As a result, the overall PCE is even decreased compared with that of the IT-4F-based device. These results pose a large challenge for broadening the optical absorption of chlorinated acceptors while maintaining high *V*_OC_s in the OPV cells.

In this work, we report a chlorinated low bandgap acceptor BTP-4Cl by replacing the halogen atoms of the fluorinated non-fullerene acceptor Y6 (herein named BTP-4F). The chlorinated acceptor BTP-4Cl shows a redshift of ca. 20 nm in optical absorption and a ca. 100 meV downshift of the LUMO level, which are easily understood by established molecular design theories^13,40^. In the OPV cells fabricated using the same polymer donor PBDB-TF, however, the BTP-4Cl-based device yields an even higher *V*_OC_ of 0.867 V compared with that of the BTP-4F-based device (0.834 V). Detailed studies on blend films indicate that the BTP-4Cl-containing film has a higher electroluminescence quantum efficiency (EQE_EL_) (3.47 × 10^−4^) than its BTP-4F counterpart (1.40 × 10^−4^), which indicates that there is a reduced non-radiative energy loss (*E*_loss,nr_) of ~24 meV that contributes to the improved *V*_OC_. Benefiting from the simultaneously broader photo-response range and improved *V*_OC_, we record high efficiencies of 16.5% and 15.3% with active areas of 0.09 and 1 cm^2^, respectively, which are among the top values for single-junction OPV cells.

## Results

### Materials design and theoretical calculations

**Fig. 1:**
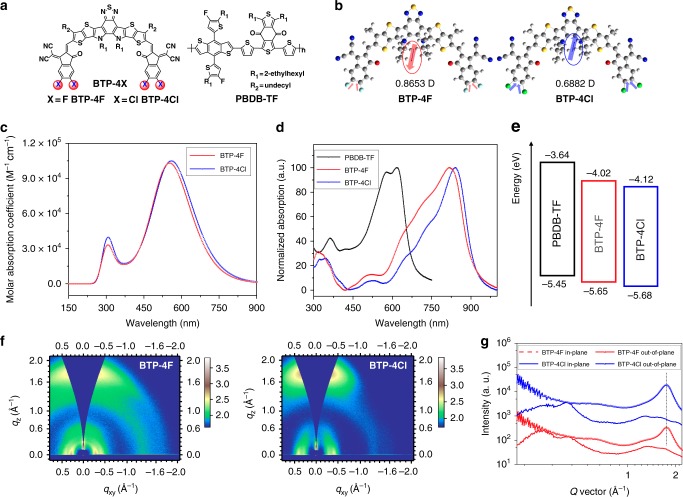
Molecular structure, optical, and electrochemical properties. **a** Chemical structure of the BTP-4X acceptors and the polymer donor PBDB-TF. **b** Molecular dipoles in the optimized molecular models for the BTP-4X acceptors. **c** Calculated UV–Vis absorption spectra of the BTP-4X. **d** Normalized UV–vis absorption spectra of the donor and acceptors as thin films. **e** Schematic energy level alignment of the materials measured by the SWV method. **f** 2D GIWAXS patterns of the neat BTP-4X films. **g** Extracted 1D profiles along the IP and OOP directions

In our previous work, we have demonstrated that chlorination has great potential for constructing OPV materials with superior performances compared to fluorination^40^. Very recently, Zou et al. reported a fluorinated non-fullerene small molecular acceptor Y6 and obtained an outstanding photovoltaic performance^21^, which motivates us to explore the applications of its chlorinated derivative in OPV cells. Figure 1a shows the molecular structures of the fluorine-containing and chlorine-containing non-fullerene acceptors and the used polymer donor PBDB-TF. As shown in Supplementary Fig. 1, the synthesis of BTP-4Cl is similar to BTP-4F in the reported literature, where the chlorine-containing terminal group was used instead of the fluorine-containing unit^21^. BTP-4Cl can be dissolved in solvents like chloroform (CF) and chlorobenzene. The detailed synthesis procedure and characterization can be found in the experimental part in the Supplementary Methods.

To study the influence of the exchange of halogen atoms on the geometries and electrical properties, we performed molecular simulations using density functional theory with the B3LYP (6–31G**) basis set, where the long alkyl side chains were simplified to methyl or ethyl groups to construct the molecular models. As displayed in Supplementary Fig. 2, the optimized molecular geometries and wavefunction distributions of the frontier orbitals including the highest occupied molecular orbits (HOMOs) and LUMOs show little difference between the two acceptors. It should be noted that from the fluorinated BTP-4F to chlorinated BTP-4Cl, the molecular energy levels of the HOMO (−5.60 to −5.65 eV) and LUMO (−3.55 to −3.63 eV) are downshifted. As the replacement of halogen atoms has a more pronounced impact on the LUMO level (80 meV, compared with 50 meV for the HOMO level), the BTP-Cl displays a bandgap that is narrowed by 30 meV compared to that of BTP-4F. These results are predictable according to the established molecular design theories, and lower *V*_OC_s are expected when applying the BTP-4Cl in OPV cells.

Unlike the acceptors with centrosymmetric features (e.g. in the case of ITIC^42^), interestingly, the BTP-4X (X represents F or Cl) molecules possess axisymmetric structures. For ITIC and analogs, although they have strong ICT effects, the overall molecular dipole moments are extremely small^37^. As presented in Fig. 1b, in comparison, the molecular dipole moments are 0.8653 and 0.6882 Debye for BTP-4F and BTP-4Cl, respectively. Since the chlorine–carbon bond has a larger dipole moment than that of the fluorine–carbon bond, the dipole direction in BTP-4Cl is turned to the opposite of that in BTP-4F. Although it is hard to relate the dipole properties to the photovoltaic performance of OPV materials, there are studies that suggest large dipoles moments are beneficial for charge separation in donor:acceptor blends^43^ and are helpful for achieving fill factors (FFs) in the devices^44^.

We conducted calculations of the excited states of BTP-4X. Supplementary Figure 3 shows the charge density distributions of the lowest excited states, from which the Coulomb attractive energies between the electrons and holes are calculated to be 2.24 and 2.21 eV for BTP-4F and BTP-4Cl, respectively. The reduced attractive energy in BTP-4Cl can be ascribed to the more delocalization of the charge density and may be beneficial for charge transfer in the donor:acceptor combinations with low-energy offsets. Figure 1c shows the calculated absorption spectra of BTP-4X, where the main peak of BTP-4Cl is redshifted by 8 nm from that of PTP-4F. The molar absorption coefficients of BTP-4X are almost the same (1.05 × 10^5^ and 1.03 × 10^5^ M^−1^ cm^−1^ for BTP-4Cl and BTP-4F, respectively).

### Optical, electrochemical, and crystalline properties

We measured the ultraviolet–visible (UV–Vis) absorption spectra of BTP-4X in diluted solutions and as thin films. As shown in Fig. 1d and Supplementary Fig. 4a, the main absorption peak was located at 732 nm for BTP-4F, while that of BTP-4Cl redshifts by 14 nm (746 nm). From solutions to films, significant redshifts of 84 and 93 nm are observed for BTP-4F and BTP-4Cl, respectively, and the main absorption bands locate in the range of 600–900 nm. The larger redshift in BTP-4Cl may be related to the stronger intermolecular π–π packing caused by the larger atomic size of chlorine and larger length of the chlorine–carbon bond. The absorption coefficients are 9.90 × 10^4^ and 1.09 × 10^5^ cm^−1^ (Supplementary Fig. 4b) for the BTP-4F and BTP-4Cl films, respectively. The broader optical absorption range and higher absorption coefficient of BTP-4Cl are beneficial for the more effective utilization of the solar photon.

We measured the electrochemical energy levels of the BTP-4X films via the square-wave voltammetry (SWV) method, and the detailed current–voltage curves are plotted in Supplementary Fig. 5. BTP-4Cl shows downshifted HOMO (by 30 meV) and LUMO (by 100 meV) levels compared to those of BTP-4F, resulting in an electrochemical bandgap that is narrowed by 70 meV (Fig. 1e). We also performed ultraviolet photoelectron spectroscopy (UPS) measurements to compare with the SWV results. As provided in Supplementary Fig. 6, BTP-4Cl has a higher ionization potential (IP) of 5.55 eV compared to BTP-4F (5.48 eV), which is consistent with the theoretical calculations.

The molecular packing properties of the acceptors were investigated by grazing-incidence wide-angle X-ray scattering (GIWAXS). From the two-dimensional (2D) patterns shown in Fig. 1f, clear π–π stacking (010) diffraction signals are observed in the out-of-plane (OOP) direction for both films, suggesting they have a preferential face-on orientations with respect to the substrate. In contrast, the peak in the BTP-4Cl film is more pronounced than that in the BTP-4F for similar film thicknesses, which may indicate a more orderly molecular packing structure. From the one-dimensional (1D) profiles extracted along the OOP direction from the 2D patterns (Fig. 1g), the π–π stacking distances are calculated to be around 3.60 Å for the BTP-4X films. These crystalline results are consistent with our previous reports^40^.

From fluorination to chlorination, the changes in the optical and electrochemical properties are highly consistent with the theoretical calculations and can be easily understood by the established molecular design theories for OPV materials. When applying the BTP-4Cl in solar cell devices, it is difficult to predict whether it will exhibit better PCEs than BTP-4F. However, higher *V*_OC_s are not expected because of the downshifted LUMO level.

### Photovoltaic performance and charge dynamics

To study the photovoltaic performance of BTP-4X, we fabricated OPV cells with an inverted configuration of indium tin oxide (ITO)/ZnO nanoparticles/PBDB-TF:BTP-4X/MoO_3_/Al, where PBDB-TF was selected as the electron donor material. First, we measured the photoluminescence (PL) spectra of the neat and blend films to investigate the photo-induced charge transfer in the donor:acceptor blend films. As displayed in Supplementary Fig. 7, the fluorescence of PBDB-TF or BTP-4X can be thoroughly quenched by the presence of the other in the corresponding blend films, suggesting that there is efficient charge transfer between the PBDB-TF and BTP-4X.

**Fig. 2:**
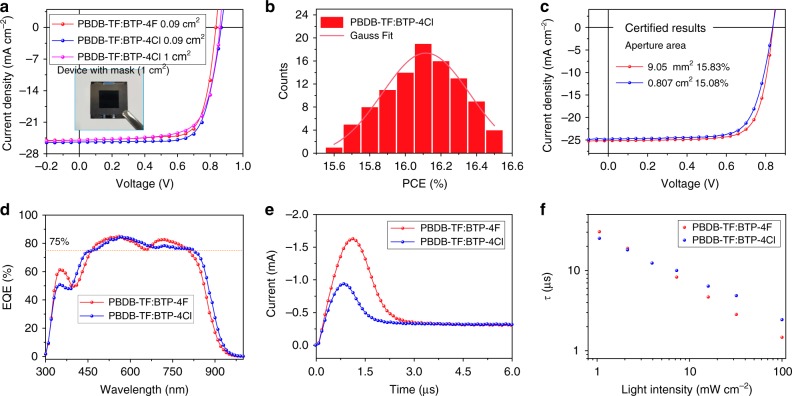
Device performance. **a** *J*−*V* curves of the PBDB-TF:IT-4X-based devices. **b** Statistical diagram of PCEs for 100 PBDB-T:BTP-4Cl-based cells. **c** *J*−*V* curves of the devices measured by the NIM, China. **d** EQE curves of the PBDB-TF:BTP-4X blend cells. **e** Photo-CELIV curves of the devices for carrier mobility calculations. **f** Carrier lifetimes under varied light intensities obtained from TPV measurements

To obtain the best device performance, we carefully optimized the fabrication conditions, including the donor:acceptor ratio, additive, and thermal annealing temperature (Supplementary Table 1). Figure 2a shows the current density–voltage (*J–V*) curves, and the detailed parameters are collected in Table 1. The PBDB-TF:BTP-4F-based device yields a good PCE of 15.6%, with a *V*_OC_ of 0.834 V, a *J*_SC_ of 24.9 mA cm^−2^, and a FF of 0.753, which is close to value in the reported literature^21^. For the device based on BTP-4Cl as the acceptor, the *V*_OC_ unexpectedly increase to 0.867 V, which is 33 mV higher than that of the BTP-4F-based device. The *J*_SC_ and FF are 25.4 mA cm^−2^ and 0.750, respectively. Overall, an outstanding PCE of 16.5% is recorded for the PBDB-TF:BTP-4Cl-based device, which represents the top result for single-junction OPV cells and is much higher than the results obtained by non-halogenated^45^ and fluorinated acceptors^21^. Figure 2b displays the statistical diagram of the efficiencies of 100 devices, and the average value reaches 16.1%. To carefully evaluate the high PCE, we sent the best device to the National Institute of Metrology, China (NIM) for certification and got a PCE of 15.83% (Fig. 2c and Supplementary Fig. 8). We fabricated the PBDB-TF:BTP-4Cl devices with varied active layer thicknesses from 70 to 300 nm. As shown in Supplementary Fig. 9 and Supplementary Table 2, we can find that the devices can deliver above 14% PCEs from 100 to 250 nm.

**Table 1:** Detailed photovoltaic parameters of the OPV cells

Devices	*V*_OC_ (V)	*J*_SC_ (mA cm^–2^)	FF	PCE (%)	Area (cm^2^)
PBDB-TF:BTP-4F	0.834 (0.833 ± 0.002)	24.9 (24.8 ± 0.2)	0.753 (0.741 ± 0.011)	15.6 (15.3 ± 0.2)	0.09
PBDB-TF:BTP-4Cl	0.867 (0.866 ± 0.002)	25.4 (25.2 ± 0.2)	0.750 (0.737 ± 0.017)	16.5 (16.1 ± 0.2)	0.09
PBDB-TF:BTP-4Cl	0.859 (0.857 ± 0.002)	25.0 (24.9 ± 0.3)	0.713 (0.694 ± 0.024)	15.3 (14.8 ± 0.3)	1.00

The average parameters were calculated from more than 30 independent cells

The photovoltaic performance discussed above is based on cells with a small active area of 9 mm^2^. As large-area fabrication of the OPV cells is of great significance for practical applications, we also fabricated PBDB-TF:BTP-4Cl devices with a 1 cm^2^ area. As presented in the *J–V* curve (the inset in Fig. 2a shows the real device) and summarized in Table 1, the device yields an impressive PCE of 15.3%. The result was also certified by NIM using a 0.807 cm^2^ mask, and a PCE of 15.08% is recorded (Fig. 2c and Supplementary Fig. 8). We noted that the best results for the devices with similar areas were only around 12–13% in earlier published reports^12,22^.

Figure 2d displays the external quantum efficiency (EQE) curves of the best cells. Both devices exhibit EQEs of over 75% in the range of 500–800 nm, and the maximum EQE values are close to 85%. By comparison, the BTP-4Cl-containing device has a broader photo-response range by approximately 15 nm than the device based on BTP-4F as the acceptor, which should be ascribed to the redshift of the optical absorption of BTP-4Cl. The integrated *J*_SC_s from the EQE spectra are calculated to be 24.7 and 25.3 mA cm^−2^, which are very close to the values obtained from *J–V* measurements.

We measured the light intensity dependence of the *J–V* characteristics to understand the recombination kinetics of the devices. As shown in Supplementary Fig. 10, by fitting the curves, we can find that both devices possess weak bimolecular and trap-assisted recombination, which may be related to the efficient charge transport in the devices (Supplementary Table 3)^46^. We measured the mobilities of the faster carrier components via photo-induced charger-carrier extraction in linearly increasing voltage (photo-CELIV) measurements^47^. From the curves shown in Fig. 2e, the mobilities are calculated to be 7.45 × 10^−5^ and 1.82 × 10^−4^ cm^2^ V^−1^ S^−1^ for the BTP-4F-containing and BTP-4Cl-containing cells, respectively. We subsequently conducted transient photovoltage (TPV) measurements to investigate the charge carrier lifetimes (*τ*). As shown in Fig. 2f, the results suggest that the PBDB-TF:BTP-4Cl-based device exhibits a slightly longer *τ* (2.4 µs) than the PBDB-TF:BTP-4F-based device (1.5 µs), which may help to obtain the high *J*_SC_ and FF of the device at such a low-energy loss^48,49^.

### Blend morphology characterization

**Fig. 3:**
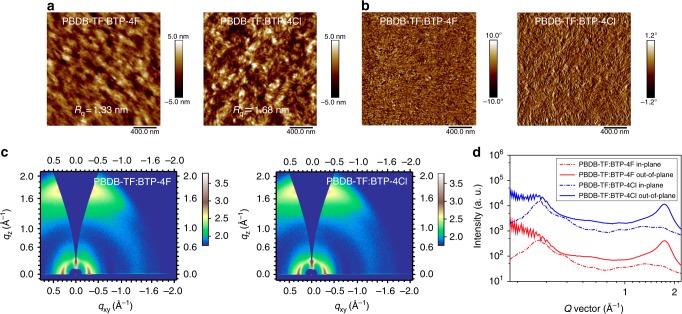
Morphology characterizations of the PBDB-TF:BTP-4X blend films. **a** AFM height images. **b** AFM phase images. **c** 2D GIWAXS patterns. **d** 1D plots extracted from the 2D patterns along the OOP and IP directions

We carried out morphology characterizations of the blend films via atomic force microscopy (AFM), transmission electron microscopy (TEM), and GIWAXS. As shown in the height images in Fig. 3a, both films have smooth surfaces. The mean-square surface roughness (*R*_q_) of the PBDB-TF:BTP-4Cl film is 1.68 nm, which is slightly higher than that of the PBDB-TF:BTP-4F film (1.33 nm). The AFM phase images (Fig. 3b) and TEM patterns (Supplementary Fig. 11) suggest that both films form nanoscale phase-separated morphologies with appropriate domain sizes in the surface and bulk. Figure 3c shows the 2d patterns from the GIWAXS measurements. The acceptors maintained their previous orientations from the blended films after blending the polymer donor PBDB-TF. Calculated from the 1D profiles, the (010) coherence lengths are around 2.0 nm for the blend films (Fig. 3d and Supplementary Table 4).

### Non-radiative energy loss

To investigate the reasons behind the unusual increase of the *V*_OC_, we studied the detailed energy losses in both devices (Table 2). According to the reported method^6,50^, the total energy loss (Δ*E*) can be divided into three parts: (1) Δ*E*_1_, radiative recombination loss above the bandgap; (2) Δ*E*_2_, radiative recombination loss below the bandgap; and (3) Δ*E*_3_, non-radiative energy loss, also called *E*_loss,nr_. First, we estimated the optical bandgaps (*E*_g_) by the intersections between the absorption and emission of the low bandgap BTP-4X^50^. Extracted from the plots shown in Supplementary Fig. 12, the *E*_g_s are calculated to be 1.407 and 1.400 eV.

**Table 2:** Detailed *V*_OC_ losses of the PBDB-TF:BTP-4X-based OPV cells

Devices	*E*_g_ (eV)	Δ*E* (eV)	$qV_{{\mathrm{OC}}}^{{\mathrm{SQ}}}$ (eV)	Δ*E*_1_ (eV)	Δ*E*_2_ (eV)	Δ*E*_3_ (eV)
PBDB-TF:BTP-4F	1.407	0.573	1.143	0.264	0.074	0.230
PBDB-TF:BTP-4Cl	1.400	0.533	1.137	0.263	0.065	0.206

**Fig. 4:**
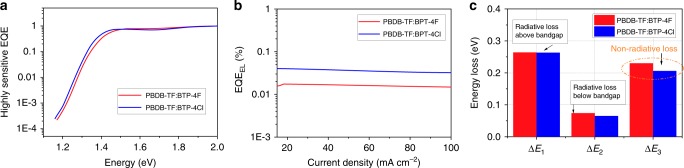
Energy loss. **a** Highly sensitive EQE curves of both devices. **b** EL quantum efficiencies of the solar cells at various injected current densities. **c** Radiative and non-radiative energy losses in the OPV cells

Based on the Shockley–Queisser (SQ) theory^51^, both devices exhibit similar values of Δ*E*_1_ of about 0.263 eV. Therefore, the SQ limit output voltages $\left( {V_{{\mathrm{OC}}}^{{\mathrm{SQ}}}{\mathrm{s}}} \right)$ are estimated to be 1.143 and 1.137 V for the devices based on BTP-4F and BTP-4Cl, respectively. We then measured the highly sensitive EQE spectra of the devices to evaluate the Δ*E*_2_. As shown in Fig. 4a, the blend films of PBDB-TF:BTP-4X have very similar sensitive-EQE spectra to the neat acceptors (Supplementary Fig. 13). In addition, the measured electroluminescence (EL) spectra of the blend films are also quite similar to the corresponding neat acceptors without additional emission peaks from the charge-transfer states. This phenomenon is commonly observed in highly efficient donor:acceptor systems with low-energy offsets and is beneficial for reducing Δ*E*_2_. When compared with the PBDB-TF:BTP-4F device, the band edge of PBDB-TF:BTP-4Cl is more abrupt, leading to a slightly reduced Δ*E*_2_ of 0.065 eV (0.074 eV for BTP-4F-containing device).

By measuring the EQE_EL_ of the devices, the third part of energy loss, Δ*E*_3_, can be evaluated using the following equation: ${\mathrm{\Delta }}E_3 = - kT\,{\mathrm{ln}}\,({\mathrm{EQE}}_{{\mathrm{EL}}})$^52^. As shown in Fig. 4b, both devices display high EQE_EL_ values with magnitudes of 10^−4^, which are very high values among the highly efficient OPV systems^50,53^. The PBDB-TF:BTP-4Cl-based device shows a higher EQE_EL_ of 3.47 × 10^−4^ than the PBDB-TF:BTP-4F-based device (1.40 × 10^−4^) and the calculated Δ*E*_3_ is 0.206 eV for the device based on PBDB-TF:BTP-4Cl, which is lower than that in the PBDB-TF:BTP-4F-based cell by 24 meV (Fig. 4c). Compared with PBDB-TF:BTP-4F blend, the lower Δ*E*_3_ in PBDB-TF:BTP-4Cl blend may be associated with its lower reorganization energy instead of the higher EQE_EL_ of the BTP-4Cl (Supplementary Fig. 14)^54,55^. The reduced non-radiative energy loss should be the major contribution to the *V*_OC_ increase in the PBDB-TF:BTP-4Cl-based device. The higher EQE_EL_ of BTP-4Cl-containing combinations is also observed in other systems when blending different polymer donors (Supplementary Fig. 15 and Supplementary Table 5).

## Discussion

To summarize, we report a chlorinated non-fullerene acceptor BTP-4Cl and achieve record PCEs of 16.5% and 15.3% for OPV cells with 0.09 and 1 cm^2^ active areas, respectively. The chlorination method broads the optical absorption and helps to obtain a high *J*_SC_ of 25.4 mA cm^−2^. Although the BTP-4Cl shows a downshifted LUMO level compared to its fluorinated analog BTP-4F, an unexpected higher *V*_OC_ of 0.867 V is obtained at a bandgap of 1.400 eV, and the corresponding energy loss is only 0.533 eV. The EQE_EL_ measurements indicate that the BTP-4Cl-based device displays a high EQE_EL_ of 3.47 × 10^−4^. Therefore, the calculated non-radiative energy loss is as low as 0.206 eV, contributing to the increase in *V*_OC_. Our work presents an example where an extended optical absorption and improved output voltage can be achieved simultaneously by the molecular design of chlorination. These results imply that the non-radiative energy loss in OPV cells can be modified by chemical modification of the photoactive materials, which provides opportunities to design of highly efficient OPV materials with low bandgap–voltage offsets.

## Methods

### Materials

The polymer donor PBDB-TF and the acceptor BTP-4F (Y6) were synthesized via referencing the reported literatures^21,56,57^. The synthesis of BTP-4Cl was synthesized by replacing the fluorinated electron-accepting unit with its chlorinated analog reported in our previous work^40^. The detailed synthetic procedures and characterizations of the chemical structures can be found in Supplementary Information.

### Fabrication and measurement of OPV cell

PBDB-TF:BTP-4X-based OPV cells were fabricated on precleaned ITO glass substrates. ZnO nanoparticles were spin-coated on the ITO with a thickness of 20 nm and baked at 100 °C for 10 min. The PBDB-TF:BTP-4F (1:1.2) blends were fully dissolved in CF at a total weight concentration of 19.8 mg mL^−1^. Before the spin-coating, 0.5% volume 1-chloronaphthalene (CN) was used as the solvent additive. As the spin-coated PBDB-TF:BTP-4Cl film from CF solution was grainy, we changed the processing solvent to chlorobenzene/CN. The optimized donor:acceptor ratio is 1:1 for PBDB-TF:BTP-4Cl. PBDB-TF:BTP-4Cl was dissolved at a total weight concentration of 20 mg mL^−1^ at 80 °C. The active layer thicknesses were controlled at 100 ± 10 nm. The blend films were treated with the thermal annealing at 100 °C for 10 min. Finally, MoO_3_/Al (10/100 nm) was evaporated onto the active layer under high vacuum. The *J–V* tests were carried out using the solar simulator (SS-F5-3A, Enlitech) in glove box. The radiative intensity (AM 1.5 G spectrum, 100 mW cm^−2^) was calibrated by the standard silicon solar cell (SRC-2020), which was calibrated by the National Institute of Metrology (NIM), China. *J−V* curves were measured in the forward direction from −0.5 to 1.5 V, with a scan step of 50 mV and a dwell time of 5 ms. The active area was defined by a metal mask with an aperture. Area of the aperture is 0.09 and 1 cm^2^ in our laboratory. Area of the aperture is 0.0905 and 0.807 cm^2^ in NIM. EQE spectra were measured using the integrated system (QE-R, Enlitech).

### Theoretical simulation

We optimized the molecular geometries of BTP-4X by Gaussian 09^58^. The hole–electron attractive energy was conducted by a wavefunction software Multiwfn^59^.

### UV–vis absorption, PL, and molecular energy level measurements

Absorption spectra of the materials were measured on a Hitachi UH5300 spectrophotometer. PL spectra were obtained on a FLS1000 PL spectrometer. The SWV measurements were conducted on a CHI650D electrochemical workstation, where the tetra-butylammonium hexafluorophosphate acetonitrile solution was used as the electrolyte and the scan rate was 100 mV s^−1^.

### Highly sensitive EQE and EQE_EL_ measurements

Highly sensitive EQE was measured using a integrated system (PECT-600, Enlitech), where the photocurrent was amplified and modulated by a lock-in instrument. EQE_EL_ measurements were performed by applying external voltage/current sources through the devices (ELCT-3010, Enlitech). All of the devices were prepared for EQE_EL_ measurements according to the optimal device fabrication conditions. EQE_EL_ measurements were carried out from 0 to 2 V).

### Charge carrier mobility and TPV measurements

Photo-CELIV mobilities and TPV data were obtained by the all-in-one characterization platform, Paios (Fluxim AG, Switzerland). The space charge-limited current method was used to estimate the hole and electron mobilities, where single-carrier devices with structures of ITO/PEDOT:PSS/active layer/Au or ITO/ZnO/active layer/PFN-Br devices were fabricated, respectively.

### AFM, TEM, and GIWAXS characterizations

AFM height and phase images were recorded on a Nanoscope V AFM microscope (Bruker), where the tapping mode was used. TEM patterns were acquired on a JEOL 2200FS instrument (bright-field mode, accelerating voltage, 200 kV). GIWAXS measurements were performed on a XEUSS SAXS/WAXS system (XENOCS, France) at the National Center for Nanoscience and Technology (NCNST, Beijing).

## Supplementary information

Supplementary Information

Solar Cells Reporting Summary

## Data Availability

The relevant data are available from the authors upon reasonable request.
