# Supplementary material for: Over 16% efficiency organic photovoltaic cells enabled by a chlorinated acceptor with increased open-circuit voltages
Source: Nat Commun. 2019 Jun 7;10:2515. doi: 10.1038/s41467-019-10351-5 (PMC6555805; doi:10.1038/s41467-019-10351-5)
Supplement: Supplementary file 1 — Supplementary Information [file 41467_2019_10351_MOESM1_ESM.pdf]

SUPPLEMENTARY INFORMATION

Over 16% efficiency organic photovoltaic cells enabled by a chlorinated acceptor with increased open-circuit voltages

Yong Cui et al.

SUPPLEMENTARY FIGURES

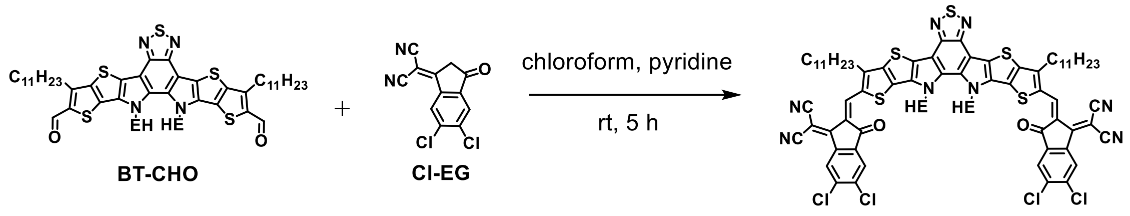

Supplementary Figure 1. The synthesis of BTP-4Cl.

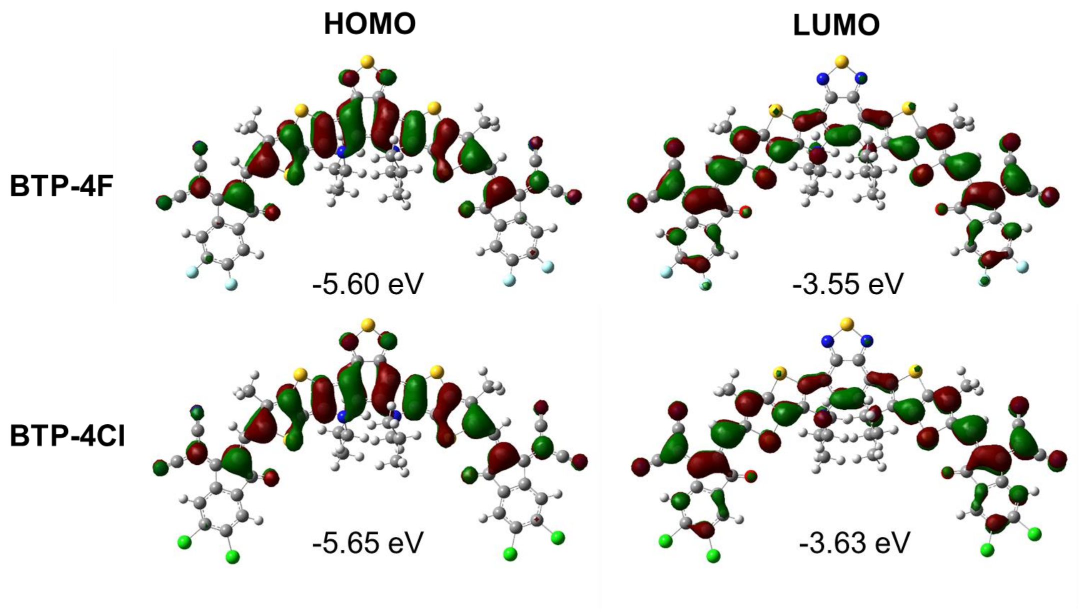

Supplementary Figure 2. Theoretical calculations of geometries, wavefunction distributions and molecular energy levels for the BTP-4X models.

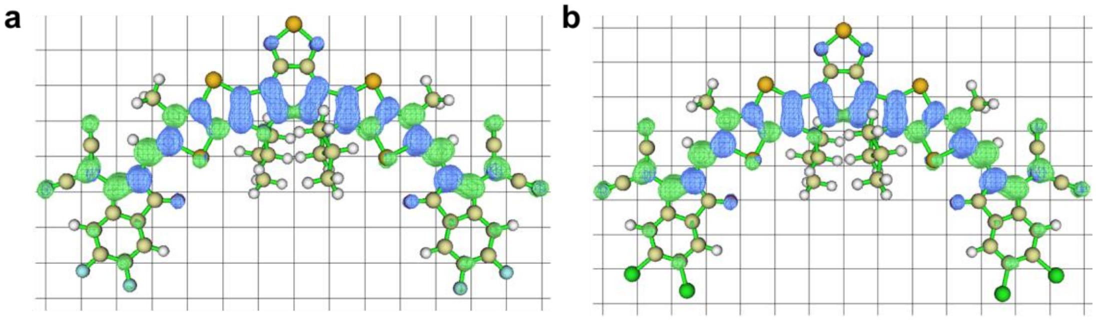

Supplementary Figure 3. The charge density distributions (hole, blue; electron, green) of the lowest excited states for **a**. BTP-4F, and **b**. BTP-4Cl.

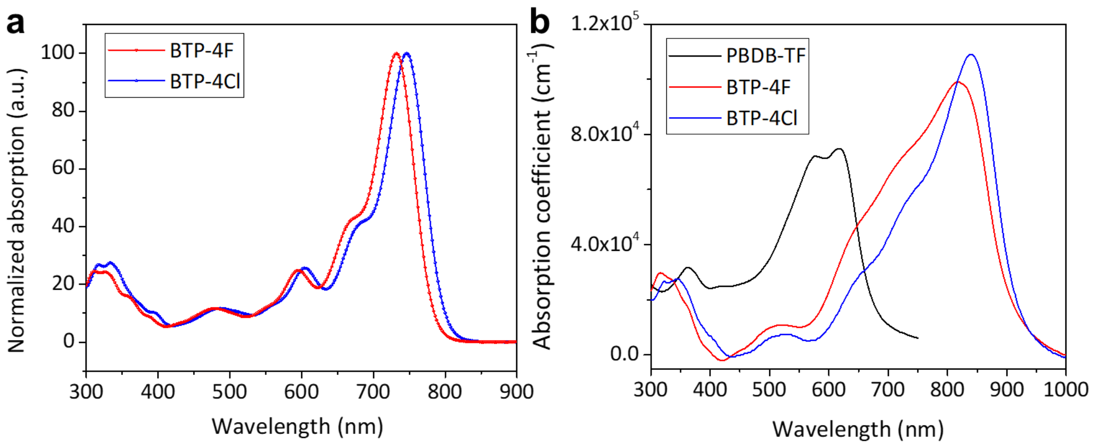

Supplementary Figure 4. a Normalized absorption spectra of the BTP-4X in diluted solutions. **b** Absorption coefficients of the BTP-4X films.

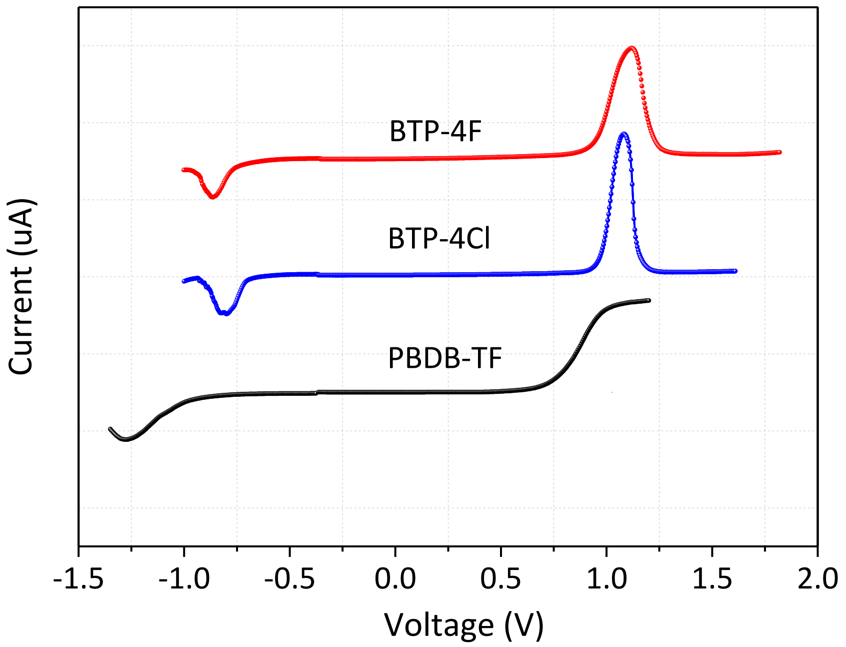

Supplementary Figure 5. Molecular energy levels determined by the SWV plots.

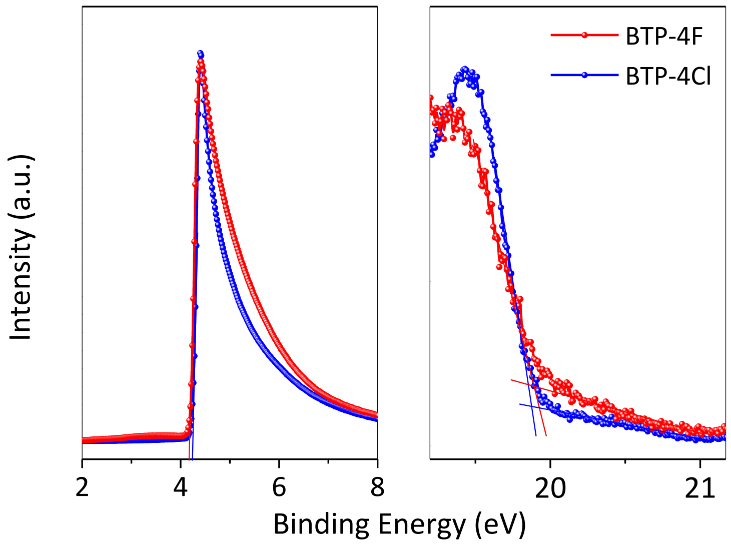

Supplementary Figure 6. UPS spectra of the acceptors: **a** the onset and **b** cutoff of the binding energy.

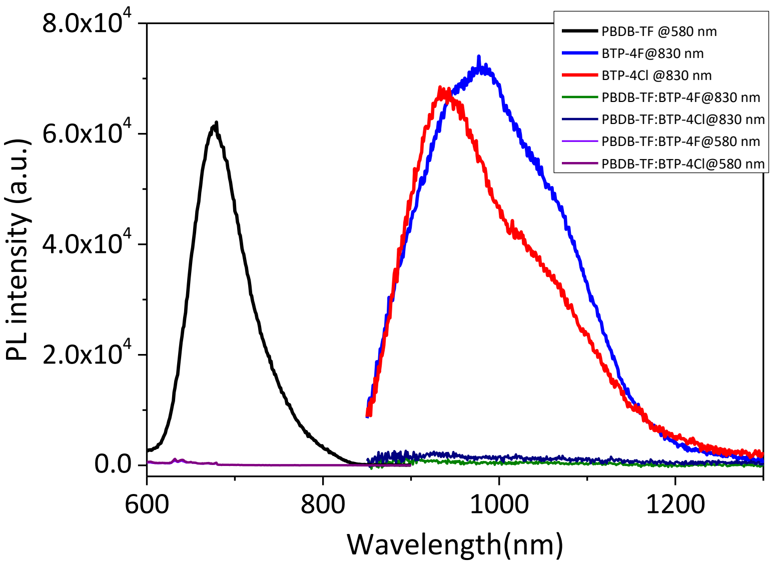

Supplementary Figure 7. PL spectra of the neat and blend films.

中国计量科学研究院

证书编号 GXtc2019-0178
Certificate No.

测 试 结 果
Calibration Results

有效面积 (mm^2)	短路电流 I_{sc}(mA)	开路电压 V_{oc}(V)	最大功率 P_{max}(mW)
9.047	2.271	0.837	1.432

最大功率电流 I_{max}(mA)	最大功率电压 V_{max}(V)	填充因子 FF (%)	转换效率(PCE) η(%)
2.076	0.690	75.36	15.83

注 Note:
1. 测试所用 mask 的面积为 9.047 mm^2（证书编号：CDjc 2016-3338）。
The mask area is 9.047 mm^2 (Certificate No.: CDjc 2016-3338).
2. 此数据仅对被测样品当时状态有效。
The data apply only at the time of the test for the sample.
（以下空白）

声明 Statement:
1.我院仅对加盖"中国计量科学研究院校准专用章"的完整证书负责。
NIM is ONLY responsible for the complete certificate with the calibration stamp of NIM.
2.本证书的测试结果仅对所校准的计量器具有效。
The certificate is ONLY valid for the tested instrument.
3.本证书用中英文两种语言表达，准确含义以中文为准。
The certificate is reported in both English and Chinese, with the Chinese version as standard.

测 试 员： [illegible]　　核 验 员： [illegible]

中国计量科学研究院

证书编号 GXtc2019-0179
Certificate No.

测 试 结 果
Calibration Results

有效面积 (mm^2)	短路电流 I_{sc}(mA)	开路电压 V_{oc}(V)	最大功率 P_{max}(mW)
80.688	19.99	0.835	12.17

最大功率电流 I_{max}(mA)	最大功率电压 V_{max}(V)	填充因子 FF (%)	转换效率(PCE) η(%)
18.17	0.670	72.91	15.08

注 Note:
1. 测试所用 mask 的面积为 80.688 mm^2（证书编号：CDjc 2016-3338）。
The mask area is 80.688 mm^2 (Certificate No.: CDjc 2016-3338).
2. 此数据仅对被测样品当时状态有效。
The data apply only at the time of the test for the sample.
（以下空白）

声明 Statement:
1.我院仅对加盖"中国计量科学研究院校准专用章"的完整证书负责。
NIM is ONLY responsible for the complete certificate with the calibration stamp of NIM.
2.本证书的测试结果仅对所校准的计量器具有效。
The certificate is ONLY valid for the tested instrument.
3.本证书用中英文两种语言表达，准确含义以中文为准。
The certificate is reported in both English and Chinese, with the Chinese version as standard.

测 试 员： [illegible]　　核 验 员： [illegible]

中国计量科学研究院

Appendix: Summary of Certificate

NIM Certificate No.: GXtc2019-0178
DUT S/N: 18-NIM-S01-F-01
Date of Test: 01/25/2019
Manufacturer: Institute of Chemistry, CAS / Hou Jianhui Group
Type: ITO/ZnO/PBDB-TF:BTP-4Cl/MoO_x/Al
Temperature Sensor/Control System: None
Mask: An aperture area of 9.047 mm^2 (Certificate No.: CDjc 2016-3338)
Environmental conditions at the time of calibration: (21.6±1) ℃, RH (16.6±2) %

The calibration has been conducted by the PV Metrology Lab of NIM (National Institute of Metrology, China). Measurement of irradiance intensity and all other measurements are traceable to the International System of Units (SI). The performance parameters reported in this certificate apply only at the time of the test for the sample.

I_{sc} [mA]	2.271	V_{oc} [V]	0.837	P_{max} [mW]	1.432
I_{max} [mA]	2.076	V_{max} [V]	0.690	Efficiency [%]	15.83
FF [%]	75.36	Area [mm^2]	9.047		

I-V Characterization Methods:
Refer to IEC60904-1 2017: Measurement of photovoltaic current-voltage characteristics
According to JJF 1622-2017: Calibration Specification of Solar Cells: Photoelectric Properties

Secondary Reference Cell:
Device S/N: 81#
Device Material: Mono-Si

Solar Simulator:
Classification: AAA (Double-light source: Xeon and Halogen)
Total irradiance: 1000 W/m^2 based on I_{sc} of the above Secondary Reference Cell.

Issue Date	01/25/2019

中国计量科学研究院

Appendix: Summary of Certificate

NIM Certificate No.: GXtc2019-0179
DUT S/N: 39-NIM-S07-F-02
Date of Test: 01/25/2019
Manufacturer: Institute of Chemistry, CAS / Hou Jianhui Group
Type: ITO/ZnO/PBDB-TF:BTP-4Cl/MoO_x/Al
Temperature Sensor/Control System: None
Mask: An aperture area of 9.047 mm^2 (Certificate No.: CDjc 2016-3338)
Environmental conditions at the time of calibration: (21.6±1) ℃, RH (16.6±2) %

The calibration has been conducted by the PV Metrology Lab of NIM (National Institute of Metrology, China). Measurement of irradiance intensity and all other measurements are traceable to the International System of Units (SI). The performance parameters reported in this certificate apply only at the time of the test for the sample.

I_{sc} [mA]	19.99	V_{oc} [V]	0.835	P_{max} [mW]	12.17
I_{max} [mA]	18.17	V_{max} [V]	0.670	Efficiency [%]	15.08
FF [%]	72.91	Area [mm^2]	80.688		

I-V Characterization Methods:
Refer to IEC60904-1 2017: Measurement of photovoltaic current-voltage characteristics
According to JJF 1622-2017: Calibration Specification of Solar Cells: Photoelectric Properties

Secondary Reference Cell:
Device S/N: 81#
Device Material: Mono-Si

Solar Simulator:
Classification: AAA (Double-light source: Xeon and Halogen)
Total irradiance: 1000 W/m^2 based on I_{sc} of the above Secondary Reference Cell.

Issue Date	01/25/2019

Supplementary Figure 8. Certification of the photovoltaic efficiency of the devices.

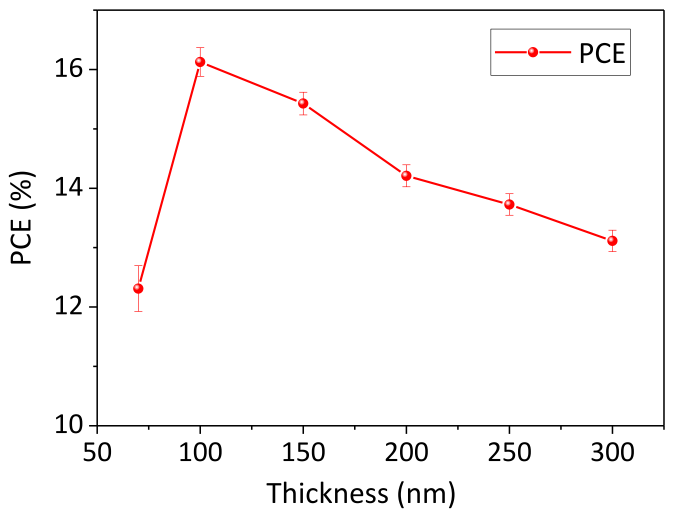

Supplementary Figure 9. PCEs of PBDB-TF:BTP-4Cl-based devices at varied active layer thicknesses.

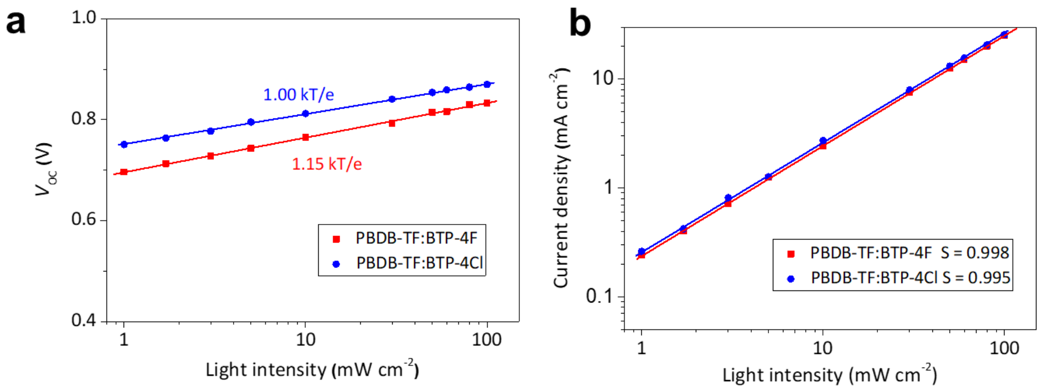

Supplementary Figure 10. V_{OC} and J_{SC} dependences on the light intensity.

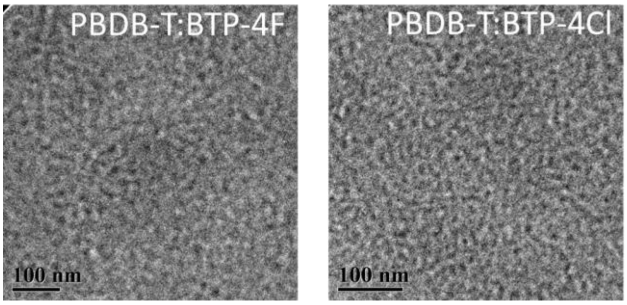

Supplementary Figure 11. TEM images of the blend films for PBDB-TF:BTP-4X.

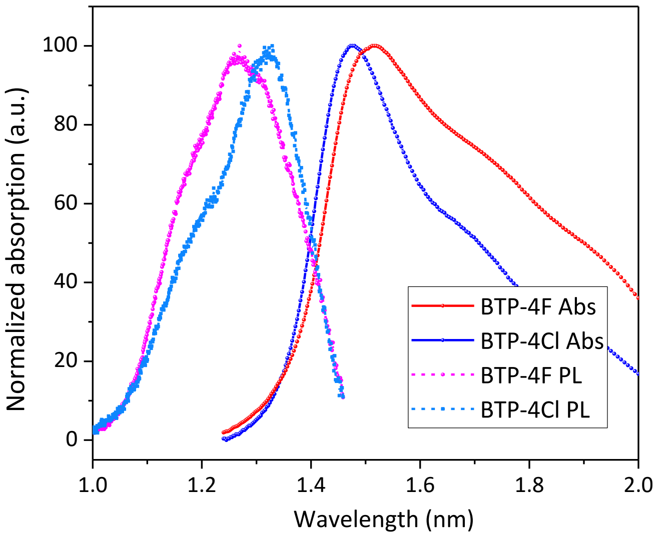

Supplementary Figure 12. Absorption and PL spectra of low bandgap BTP-4X acceptors.

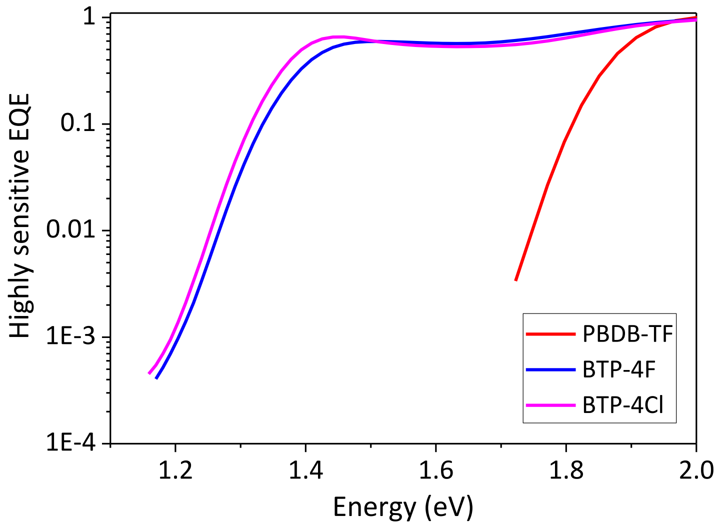

Supplementary Figure 13. Highly senseitve EQE curves of neat materials.

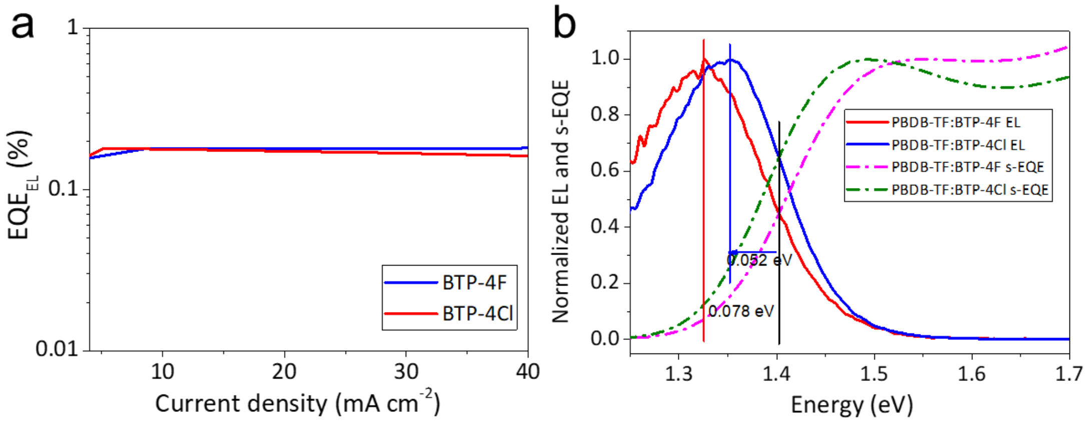

Supplementary Figure 14. **a**. EQE_{EL} of the neat acceptor films. **b**. Normalized EL and s-EQE of the blend films.

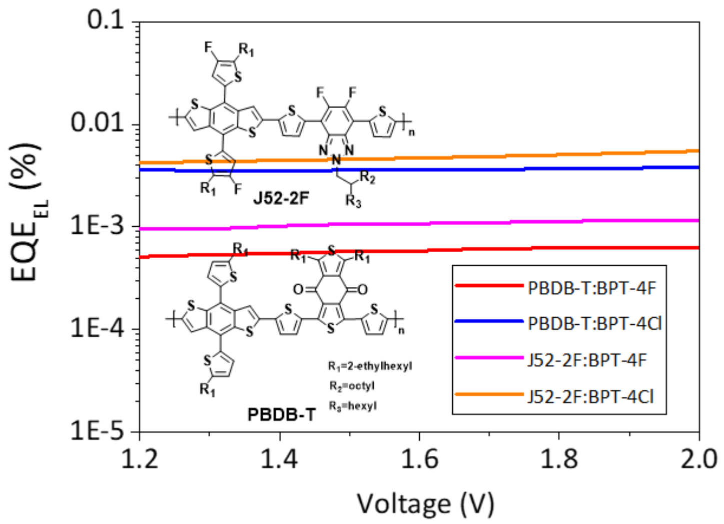

Supplementary Figure 15. The EQE_{EL} of the different blend combinations based on polymer donors of PBDB-T or J52-2F.

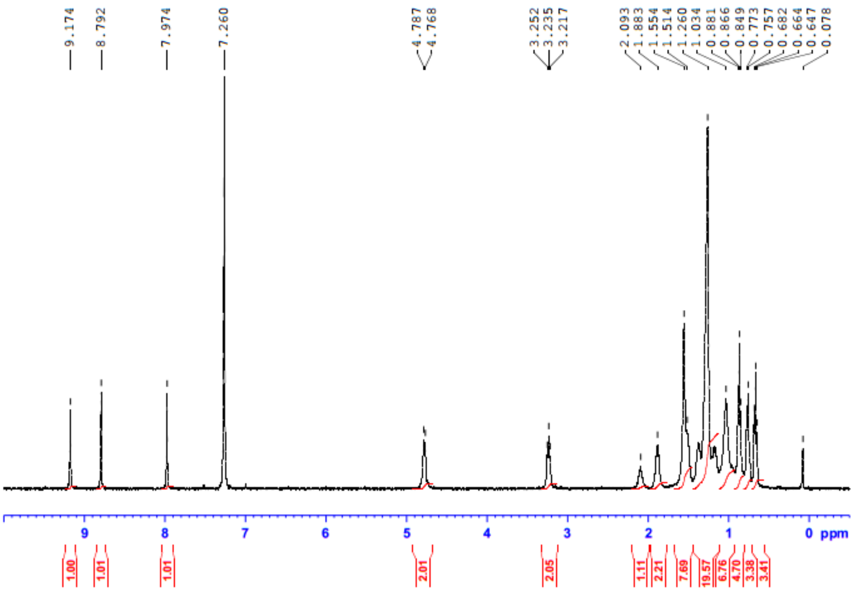

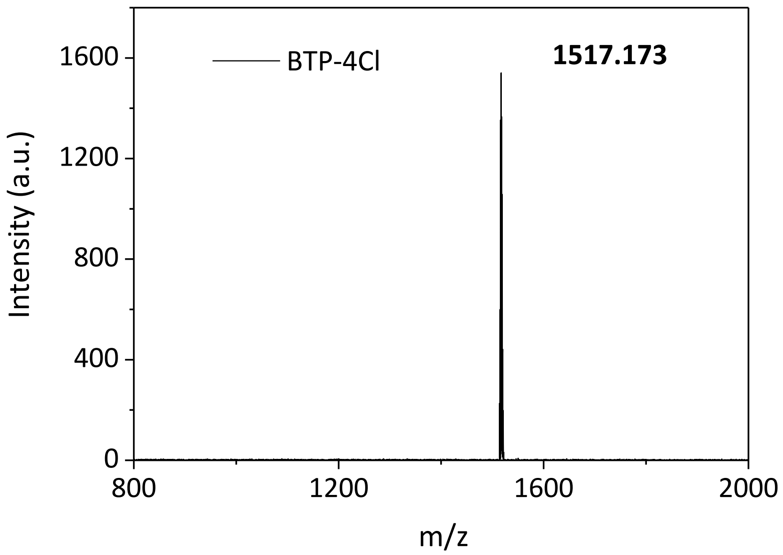

Supplementary Figure 16. [1] NMR and MS-MALDI spectra of the BTP-4Cl.

SUPPLEMENTARY TABLES

Supplementary Table 1. Detailed photovoltaic parameters of the PBDB-TF:BTP-4Cl-based devices processed by varied conditions.

1) To get the best D/A ratio, 0.5 v/v of CN is used as the solvent additive, the films are thermally annealed at 100 ^{o}C for 10 min.

D/A	V_{OC} (V)	J_{SC} (mA cm^{-2})	FF (%)	PCE (%)
1.2:1	0.869	24.6	73.8	15.8 (15.5 ± 0.2)
**1:1**	0.864	25.3	73.1	16.0 (15.7 ± 0.2)
1:1.2	0.858	25.1	72.2	15.5 (15.1 ± 0.3)

2) To get the best CN content, the D/A ratio is kept at 1:1, and the films are thermally annealed at 100 ^{o}C for 10 min.

CN v/v	V_{OC} (V)	J_{SC} (mA cm^{-2})	FF (%)	PCE (%)
without	0.869	24.2	0.641	13.5 (13.2 ± 0.2)
0.5	0.864	25.3	0.731	16.0 (15.7 ± 0.2)
1	0.865	25.3	0.742	16.2 (15.8 ± 0.2)
**2**	0.867	25.4	0.750	16.5 (16.1 ± 0.2)
3	0.864	24.1	0.752	15.7 (15.1 ± 0.4)

3) The following is the optimization of the thermal annealing temperature, where D/A ratio is 1:1, CN v/v is 2%.

Temperature	V_{OC} (V)	J_{SC} (mA cm^{-2})	FF (%)	PCE (%)
80℃	0.870	25.2	0.741	16.2 (15.9 ± 0.2)
**100℃**	0.867	25.4	0.750	16.5 (16.1 ± 0.2)
120℃	0.859	25.5	0.739	16.2 (15.8 ± 0.2)
140℃	0.853	24.7	0.728	15.3 (15.0 ± 0.2)

Supplementary Table 2. Detailed photovoltaic parameters of the PBDB-TF:BTP-4Cl-based devices at varied active layer thicknesses.

Thickness (nm)	V_{OC} (V)	J_{SC} (mA cm^{-2})	FF	PCE (%)
70 ± 5	0.865	20.7	0.721	12.9 (12.3 ± 0.4)
100 ± 4	0.867	25.4	0.750	16.5 (16.1 ± 0.2)
150 ± 6	0.866	25.6	0.714	15.8 (15.4 ± 0.2)
200 ± 6	0.861	26.1	0.644	14.5 (14.2 ± 0.2)
250 ± 10	0.857	26.7	0.613	14.0 (13.7 ± 0.2)
300 ± 8	0.854	27.2	0.578	13.4 (13.1 ± 0.2)

Supplementary Table 3. The charge mobilities of the blend measured by SCLC method.

Blend	hole mobility μ_h (cm^2 V^{-1} s^{-1})	electron mobility μ_e (cm^2 V^{-1} s^{-1})	μ_h/μ_e
PBDB-TF:BTP-4F	1.25×10^{-4}	7.33×10^{-5}	1.7
PBDB-TF:BTP-4Cl	2.12×10^{-4}	1.58×10^{-4}	1.3

Supplementary Table 4. The coherence length (CL) of (010) peaks for the films.

Materials	(010) Peak position (Å^{-1})	(010) FWHM	(010) CL (nm)
BTP-4F	1.75	0.28	2.02
BTP-4Cl	1.75	0.27	2.09
PBDB-TF: BTP-4F	1.75	0.28	2.02
PBDB-TF: BTP-4Cl	1.75	0.28	2.02

Supplementary Table 5. Detailed photovoltaic parameters of the devices based on different polymer donors (PBDB-T and J52-2F) and BTP-4X acceptors.

Devices	V_{OC} (V)	J_{SC} (mA cm^{-2})	FF	PCE (%)	$\triangle E_3$(eV)
J52-2F:BTP-4F	0.770	25.4	0.679	13.3 (13.0 ± 0.2)	0.294
J52-2F:BTP-4Cl	0.797	25.8	0.700	14.4 (14.1 ± 0.2)	0.254
PBDB-T:BTP-4F	0.711	25.1	0.626	11.2 (10.8 ± 0.2)	0.318
PBDB-T:BTP-4Cl	0.774	25.5	0.609	12.0 (11.6 ± 0.3)	0.265

Supplementary Method.

Material. The core unit of BT-CHO was synthesized by referencing the reported literature.[1,2] The electron-accepting unit was synthesized in our previous work.[3,4] Other reagents and solvents used were purchased from commercial sources and used as received.

Synthesis of the BTP-4Cl.

The core compound BT-CHO (205 mg, 0.2 mmol) and the electron-accepting unit (263 mg, 1 mmol) were added to the chloroform (15 mL) under the protection of argon. Then, 0.5 mL of pyridine was added slowly. The reaction was stirred for 5 hours at room temperature. The mixture was purified by silica gel column chromatography using chloroform as the eluent. 225 mg of black solid was obtained as the product (yield 74%). MS (MALDI) *m/z*: Calcd for $C_{82}H_{86}Cl_4N_8O_2S_5$: 1517.424; Found 1517.173. Analytical calculation for $C_{82}H_{86}Cl_4N_8O_2S_5$: C, 64.89; H, 5.71; N, 7.38. Experimental result: C, 64.55; H, 5.99; N, 6.97. The ^{1}H NMR and MS spectra are provided as **Supplementary Figure 16**. As BTP-4Cl has a limited solubility in chloroform, it's hard to get clear ^{13}C NMR spectrum, therefore we don't provide it here.

Supplementary Reference

1 Yuan, J. *et al.* Single-Junction Organic Solar Cell with over 15% Efficiency Using Fused-Ring Acceptor with Electron-Deficient Core. *Joule* **3**, 1140-1151 (2019).

2 Cheng, Y.-J. *et al.* Synthesis, Photophysical and Photovoltaic Properties of Conjugated Polymers Containing Fused Donor–Acceptor Dithienopyrrolobenzothiadiazole and Dithienopyrroloquinoxaline Arenes. *Macromolecules* **45**, 2690-2698 (2012).

3 Zhang, H. *et al.* Over 14% Efficiency in Organic Solar Cells Enabled by Chlorinated Nonfullerene Small-Molecule Acceptors. *Adv. Mater.* **30**, e1800613 (2018).

4 Cui, Y. *et al.* Efficient Semitransparent Organic Solar Cells with Tunable Color enabled by an Ultralow-Bandgap Nonfullerene Acceptor. *Adv. Mater.* **29**, 1703080 (2017).
