# Supplementary material for: Over 16% efficiency organic photovoltaic cells enabled by a chlorinated acceptor with increased open-circuit voltages
Source: Nat Commun. 2019 Jun 7;10:2515. doi: 10.1038/s41467-019-10351-5 (PMC6555805; doi:10.1038/s41467-019-10351-5)
Supplement: Supplementary file 2 — Solar Cells Reporting Summary [file 41467_2019_10351_MOESM2_ESM.pdf]

nature research

Corresponding author(s): Huifeng Yao

Solar Cells Reporting Summary

Nature Research wishes to improve the reproducibility of the work that we publish. This form is intended for publication with all accepted papers reporting the characterization of photovoltaic devices and provides structure for consistency and transparency in reporting. Some list items might not apply to an individual manuscript, but all fields must be completed for clarity.

For further information on Nature Research policies, including our data availability policy, see Authors & Referees.

Experimental design

Please check: are the following details reported in the manuscript?

1. Dimensions

Area of the tested solar cells
- [x] Yes — Area of the tested solar cells is provided in methods, section"Fabrication and measurement of OPV cell"
- [ ] No

Method used to determine the device area
- [x] Yes — The method is provided in methods, section"Fabrication and measurement of OPV cell"
- [ ] No — *Explain why this information is not reported/not relevant.*

2. Current-voltage characterization

Current density-voltage (J-V) plots in both forward and backward direction
- [ ] Yes — *State where this information can be found in the text.*
- [x] No — Just J-V plot in forward direction since there is no hysteresis in organic solar cell.

Voltage scan conditions
For instance: scan direction, speed, dwell times
- [x] Yes — The scan conditions are provided in methods, section"Fabrication and measurement of OPV cell"
- [ ] No — *Explain why this information is not reported/not relevant.*

Test environment
For instance: characterization temperature, in air or in glove box
- [x] Yes — Test environment is provided in methods, section"Fabrication and measurement of OPV cell"
- [ ] No — *Explain why this information is not reported/not relevant.*

Protocol for preconditioning of the device before its characterization
- [ ] Yes — *State where this information can be found in the text.*
- [x] No — No preconditioning protocol.

Stability of the J-V characteristic
Verified with time evolution of the maximum power point or with the photocurrent at maximum power point; see ref. 7 for details.
- [ ] Yes — *State where this information can be found in the text.*
- [x] No — there is no corresponding testing equipment

3. Hysteresis or any other unusual behaviour

Description of the unusual behaviour observed during the characterization
- [ ] Yes — *State where this information can be found in the text.*
- [x] No — No. In general, organic solar cells do not have hystereisis problems.

Related experimental data
- [ ] Yes — *State where this information can be found in the text.*
- [x] No — NO.

4. Efficiency

External quantum efficiency (EQE) or incident photons to current efficiency (IPCE)
- [x] Yes — EQE curve is shown in Figure 2d
- [ ] No — *Explain why this information is not reported/not relevant.*

A comparison between the integrated response under the standard reference spectrum and the response measure under the simulator
- [ ] Yes — *State where this information can be found in the text.*
- [x] No — NO

For tandem solar cells, the bias illumination and bias voltage used for each subcell
- [ ] Yes — *State where this information can be found in the text.*
- [x] No — Our cells were only fabricated for single solar cells.

5. Calibration

Light source and reference cell or sensor used for the characterization

☒ Yes ☐ No

Relative information is provided in methods, section"Fabrication and measurement of OPV cell"

Explain why this information is not reported/not relevant.

Confirmation that the reference cell was calibrated and certified

☒ Yes ☐ No

Relative information is provided in methods, section"Fabrication and measurement of OPV cell"

Explain why this information is not reported/not relevant.

Calculation of spectral mismatch between the reference cell and the devices under test

☐ Yes ☒ No

State where this information can be found in the text.

The spectral mismatch factor was determined at National Institute of Metrology, China (NIM). We do not have the detailed information for the method.

6. Mask/aperture

Size of the mask/aperture used during testing

☒ Yes ☐ No

Size of the mask is provided in methods, section"Fabrication and measurement of OPV cell"

Explain why this information is not reported/not relevant.

Variation of the measured short-circuit current density with the mask/aperture area

☒ Yes ☐ No

Information is provided in table 2.

Explain why this information is not reported/not relevant.

7. Performance certification

Identity of the independent certification laboratory that confirmed the photovoltaic performance

☒ Yes ☐ No

Certified results are provided in the figure 2c.

Explain why this information is not reported/not relevant.

A copy of any certificate(s)
Provide in Supplementary Information

☒ Yes ☐ No

certificates are provided in the supplementary information.

Explain why this information is not reported/not relevant.

8. Statistics

Number of solar cells tested

☒ Yes ☐ No

Number of cells tested is provided in Table 1.

Explain why this information is not reported/not relevant.

Statistical analysis of the device performance

☒ Yes ☐ No

Statistical results of the devices are listed in Table 1.

Explain why this information is not reported/not relevant.

9. Long-term stability analysis

Type of analysis, bias conditions and environmental conditions
For instance: illumination type, temperature, atmosphere humidity, encapsulation method, preconditioning temperature

☐ Yes ☒ No

State where this information can be found in the text.

NO
